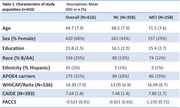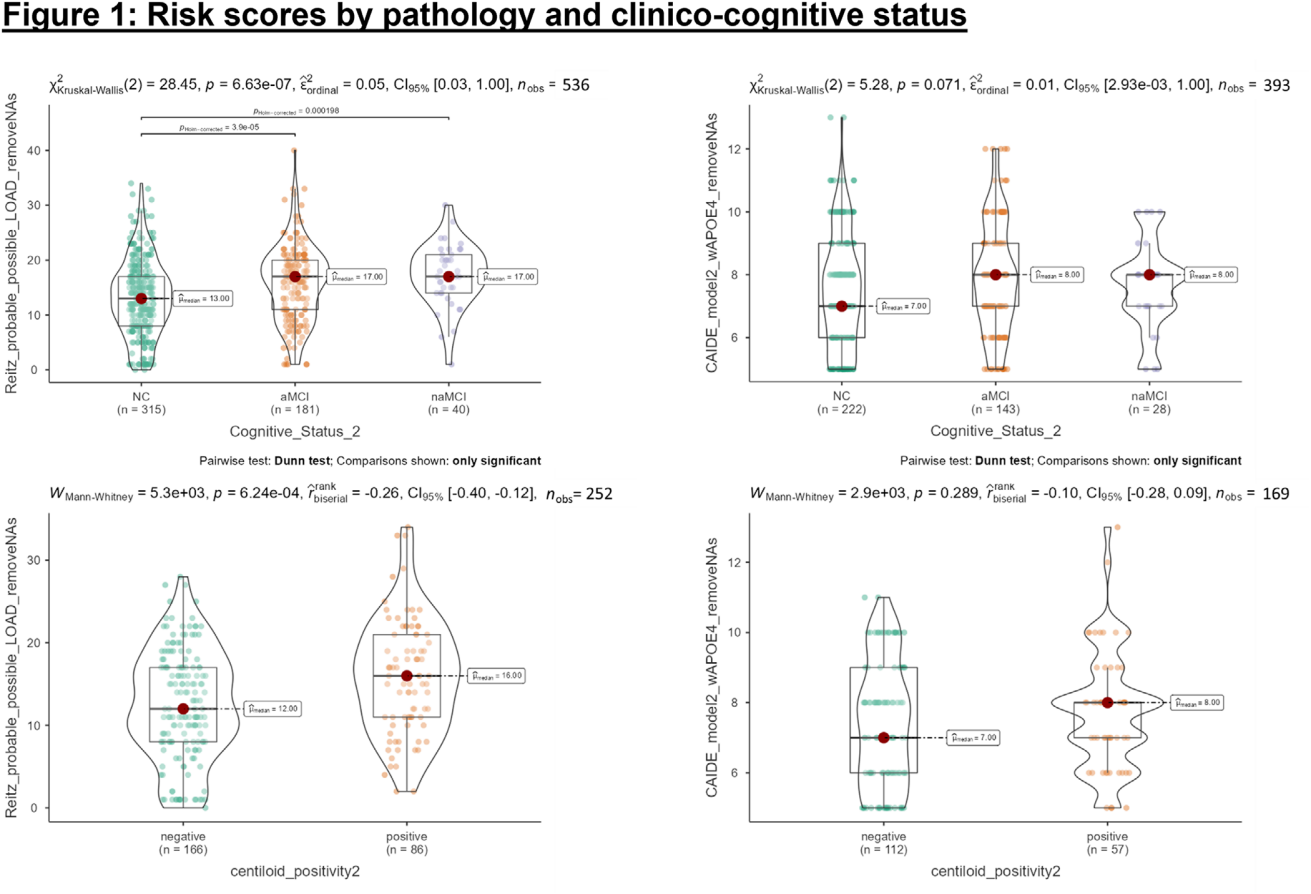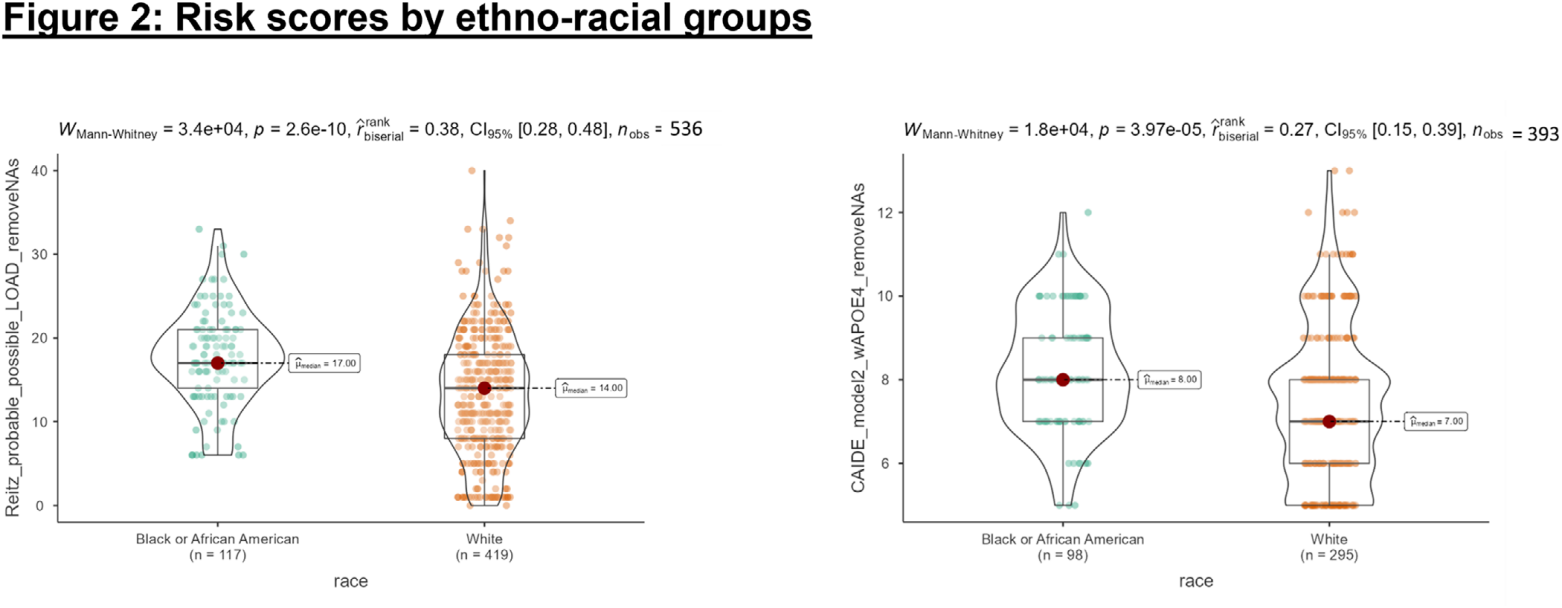# Vascular‐based risk scores for the prediction of Alzheimer’s disease pathology biomarkers and cognitive status in older adults at‐risk for dementia: Exploratory analysis of sex and ethno‐racial differences

**DOI:** 10.1002/alz.093316

**Published:** 2025-01-03

**Authors:** Chinedu Udeh‐Momoh, Tugce Duran, Timothy M. Hughes, Kiran K. Solingapuram Sai, James R. Bateman, Goldie S. Byrd, Michelle M. Mielke, Suzanne Craft, Samuel N. Lockhart

**Affiliations:** ^1^ Wake Forest University School of Medicine, Winston‐Salem, NC USA

## Abstract

**Background:**

Vascular‐based dementia risk scores (VDRS) which reliably predict risk of Alzheimer’s disease and related dementias (ADRDs), may be useful to identify at‐risk individuals for secondary prevention trials. Dementia risk scores have typically focused on predicting ADRD‐associated symptoms, with fewer studies assessing capacity for detecting individuals with underlying brain pathologies. We compare the predictive value of two vascular‐based risk scores (CAIDE and Reitz VDRSs) for discriminating AD‐related histopathological and structural abnormalities, further considering race and gender differences.

**Method:**

We included non‐demented (self‐identified) White and Black participants at the Wake Forest ADRC Clinical Core with baseline UDSv3 cognitive testing (used to calculate the Preclinical Alzheimer’s Cognitive Composite ‐ PACC5), clinical adjudication, amyloid (PiB) PET, *APOE* genotype, and metabolic characterization for diabetes including prediabetes. WHICAP/Reitz scores were calculated using components of sex, education, age, race, ethnicity, hypertension, diabetes, WHR, HDL, smoking and *APOE*‐ε4 status. CAIDE scores included age, education, sex, systolic BP, BMI, physical activity, total cholesterol, and *APOE*‐ε4. We assessed differences in VDRS among cognitive, amyloid PET and racial groups.

**Result:**

616 participants (Table 1) had baseline data available for analysis. Of 287 with amyloid PET, 33% were amyloid positive (CL>20). Median Reitz scores differed by cognitive status (Figure 1A), with higher mean scores noted for those with amnestic (aMCI) and non‐amnestic MCI (naMCI) compared to those with normal cognition (naMCI/aMCI: median 17, p<.001). Reitz scores were also significantly higher in amyloid positive vs negative participants (Figure 1C) as well as in Black vs White participants (Figure 2A) (median (IQR): 16 (2,34) vs 12 (0,28), 17 (0,33) vs 14 (0,40) respectively; p<.001), however no sex differences were noted. In contrast, we found no significant differences in CAIDE scores among cognitive and amyloid pathology groups (p>0.05, Figures 1B and 1D), except ethno‐racial groups (p<.001, Figure 2B).

**Conclusion:**

Significant differences in distinct VDRSs were found in relation to brain amyloid pathology levels and clinico‐cognitive status, with ethno‐racial influences potentially contributing to noted differences. Risk composites accounting for these factors will be useful for greater precision around pre‐selection of at‐risk populations towards targeted trials for disease modifying therapies and optimal management of clinical care.